# Habitual physical activity levels in women attending the one-stop infertility clinic: a prospective cross-sectional observational study

**DOI:** 10.1530/RAF-22-0067

**Published:** 2022-09-26

**Authors:** Nicola Tempest, Madeleine France-Ratcliffe, Hannan Al-Lamee, Evie R Oliver, Emily E Slaine, Andrew J Drakeley, Victoria S Sprung, Dharani K Hapangama

**Affiliations:** 1 Centre for Women’s Health Research, Department of Women’s and Children’s Health, Institute of Life Course and Medical Sciences, University of Liverpool, Member of Liverpool Health Partners, Liverpool, UK; 2 Liverpool Women’s NHS Foundation Trust, Member of Liverpool Health Partners, Liverpool, UK; 3 Hewitt Centre for Reproductive Medicine, Liverpool Women’s NHS Foundation Trust, Liverpool, UK; 4 Research Institute for Sport & Exercise Sciences, Liverpool John Moores University, Liverpool, UK

**Keywords:** physical activity, exercise, fertility, infertility, conception

## Abstract

Optimisation of lifestyle factors such as smoking and alcohol is encouraged to improve fecundability rates in the fertility setting. Currently, routine fertility consultations do not involve counselling or imparting advice regarding habitual physical activity (PA) and/or structured exercise, despite data showing that vigorous PA can be associated with delayed time to pregnancy. Therefore, this study aimed to determine habitual PA in a sample of women attending the one-stop infertility (OSI) clinic. In total, 250 women attending a large tertiary level NHS fertility unit prospectively completed a questionnaire anonymously over a period of 9 months. The participants (mean age: 34 ± 5 years, mean BMI: 29 ± 7 kg/m^2^) habitual PA levels varied from vigorous exercise on ≥5 days/week (8%, *n*  = 17) to no moderate or high-intensity activities across the whole week (66%, *n*  = 29). A majority of women reported no structured exercise (72%, *n*  = 179). No association was identified between any domain of PA and BMI, age, alcohol units, regular periods, or time spent trying to conceive (*P* > 0.05). The participants habitual PA levels varied widely and no association between any domain of PA and background of the women was identified. No existing evidence and/or guidelines to explicitly inform women attempting to conceive regarding recommended PA levels are available, despite PA being a modifiable, affordable, and feasible lifestyle choice with the possible potential to improve fertility. A large-scale, clinical trial assessing the effects of PA on fecundability is warranted to gain insight into the potential of this lifestyle factor to improve fertility outcomes and to explore the underlying biological mechanisms involved.

**Lay summary:**

Infertility affects approximately one in seven couples with many and varied causes, including lifestyle factors such as smoking, alcohol, and diet. Lifestyle changes are low-cost unimposing options to implement in routine fertility care. Information on regular physical activity is not currently enquired from women and no agreement regarding the best levels of exercise exists for fertility patients. In this study, we aimed to determine the exercise habits of women attending the OSI clinic. In total. 250 women attending OSI clinic over a period of 9 months completed a questionnaire collecting data on their exercise habits. The levels of physical activity performed varied widely from vigorous exercise on ≥5 days/week to no moderate or high-intensity activities across the whole week. A majority of women did no structured exercise (72%). These novel data highlight the variations in form, type and intensity of exercise women who attend OSI clinics perform. Currently, there is no existing evidence and/or guidelines to explicitly inform women attempting to conceive regarding the recommended physical activity levels. Physical activity is a modifiable, affordable, and feasible lifestyle choice that is not currently acknowledged in the fertility setting and has the potential to improve fertility.

## Introduction

Infertility is defined as a reproductive disease, described as the failure to achieve a clinical pregnancy within 12 months or more, of regular unprotected sexual intercourse (Zegers-Hochschild *et al.* 2009). Approximately one in seven couples are likely to experience difficulty conceiving (NICE 2017, NHS). The main causes often overlap and, in the UK, approximately 40% of cases identify disorders in both men and women (NICE 2017). They include unexplained infertility (25%), ovulatory disorders (25%), tubal damage (20%), factors in the male causing infertility (30%), and uterine or peritoneal disorders (10%) (NICE 2017). Infertility causes a huge burden on those couples affected by both psychological and physical strain (Schmidt 2006). Some lifestyle and recreational factors, such as alcohol/drug consumption, smoking, and hobbies, can affect fecundability rates and couples are counselled regarding the optimisation of these, accordingly (Britton 2016, NICE 2017, Ricci *et al.* 2017, Ayton 2019, Imterat *et al.* 2019, Sermondade *et al.* 2019).

Currently, routine fertility consultations do not involve counselling or imparting advice regarding habitual physical activity (PA) and/or structured exercise despite data from two large observational fecundability studies showing that vigorous PA was associated with delayed time to pregnancy in women with a normal BMI not currently seeking fertility care (Wise *et al.* 2012, McKinnon *et al.* 2016). Numerous other studies have examined the association between exercise and specific fertility outcomes, but the available evidence remains equivocal (Bullen *et al.* 1985, Ahrens *et al.* 2014, Mena *et al.* 2020). This variability within the literature holds true for women living with polycystic ovarian syndrome (PCOS), a heterogenous condition frequently associated with ovulatory disorders. It has been observed that increased PA and exercise in these women improve overall health outcomes and symptom presentation, but findings related to fertility outcomes are contradictory and relate more to weight status than PA levels (Harrison *et al.* 2011, Sprung *et al.* 2013, Kite *et al.* 2019, Stepto *et al.* 2019, Dos Santos *et al.* 2020). With this in mind, a change in habitual PA is potentially a cost-effective, minimally invasive adjunct, associated with general health benefits, for women to consider as part of their routine fertility care.

Therefore, the aim of the current study was to determine habitual PA in a sample of women attending the one-stop infertility (OSI) clinic. Furthermore, we sought to assess the willingness of these women to participate in further research examining the influence of PA on fertility outcomes.

## Materials and methods

The questionnaire was distributed to 350 non-pregnant females, aged 34 ± 5 years, who attended the OSI clinic at The Hewitt Fertility Centre, Liverpool (a specialist tertiary referral fertility centre), between November 2020 and July 2021. Eligibility was based on three simple criteria; (i) non-pregnant women; (ii) aged 18–42 years; and (iii) attending the clinic for fertility consultation.

### Ethical approval

This observational study conformed to the Declaration of Helsinki and was approved by the London-Bloomsbury Research Ethics Committee (REC: 20/PR/0458). All participants were informed of the protocol verbally and in writing prior to completion of the questionnaire. This detailed that consent is assumed on the voluntary return of the completed questionnaire (which did not contain any personal identifiable data).

### Demographics

Women who completed the International Physical Activity Questionnaire Short Form (IPAQ-SF) self-reported their age, height, weight, ethnicity, smoking status, alcohol consumption, occurrence of regular periods, gravidity, parity, length of time trying to conceive, diagnosis of fertility pathology, previous fertility treatment, medical conditions, and regular medication.

### International Physical Activity Questionnaire

The IPAQ-SF, which has been validated and successfully administered to similar groups (e.g. female clinical groups (Russo *et al.* 2018)), was identified as the most suitable, validated, clinical tool for assessing contemporaneous self-reported habitual PA and exercise at a population observation level (Craig *et al.* 2003). The IPAQ-SF requires participants to report how many days they walked and did moderate or vigorous PA for at least 10 min at a time in 7 days prior to completing the questionnaire. PA measured via the IPAQ-SF is classified as high, moderate, and low. High-level PA was defined as a vigorous activity, of at least 20 min per day, occurring on ≥3 days per week, achieving a minimum of 1500 metabolic equivalent (MET) minutes per week in total or 7 days of any combination of walking, moderate-intensity or vigorous intensity activities, achieving a minimum of 3000 MET minutes per week in total. Moderate PA was defined as any of the following: vigorous activity, of at least 20 min per day, occurring on ≥3 days per week; ≥5 days of moderate-intensity activity and/or walking of at least 30 min per day; ≥5 days of any combination of walking, moderate-intensity or vigorous intensity activities; all achieving a minimum of at least 600 MET minutes per week in total. Participants were classified as low PA if they did not achieve moderate or high levels of PA (Cheng 2016).

### Further research

Women were also asked if they would be interested in participating in a further research study examining the effects of PA on fertility outcomes.

### Statistical analysis

Initially, summary data were extracted and reported; mean and s.d. for continuous data and percentages for categorical data were used. Correlations between PA and lifestyle factors were assessed using Pearson’s correlation coefficient. The alpha level of statistical significance was set at *P* < 0.05. Statistical analysis was performed using SPSS for Windows (Version 26.0, SPSS).

## Results

A response rate of 71.4% (250/350) was observed in this study, but not all participating women completed all sections of the demographic data collection and IPAQ-SF.

### Descriptive characteristics

A total of 250 data sets were analysed from respondents (age: 34 ± 5 years, BMI: 29 ± 7 kg/m^2^) (Table 1). Sixty percent (*n* = 143) of the women never had a pregnancy, and 82% (*n* = 199) did not have children. Most women (64.4%, *n*  = 161) had no diagnosed cause for infertility (first tertiary clinic appointment and therefore most not had investigations). The common causes documented included: PCOS (15.2%, *n*  = 38), male factor (9.2%, *n*  = 23), endometriosis (3.6%, *n*  = 9), and premature ovarian failure (POF) (2.8%, *n*  = 7). 72% (*n* = 180) had no past medical history of note. Commonly reported medical co-morbidities included asthma (9.2%, *n*  = 23), depression (5.2%, *n*  = 13), migraines (3.2%, *n*  = 8), hypothyroid (2.8%, *n*  = 7), and hypertension (2%, *n*  = 5). The majority (70.4%, *n*  = 176) of women were not taking regular, prescribed medications. Among those who reported regular medication use, antidepressants (8%, *n*  = 20) or inhalers (6.4%, *n*  = 16) were most commonly listed.

**Table 1:** Participant demographics. Data are presented as *n* (%) or as mean ± s.d.

Demographic	Values
Participants, *n*	250
Age (years)	34 ± 5
BMI^*^ (kg/m^2^)	29 ± 7
Underweight (˂18.5)	1 (1%)
Normal weight (≥18.5, to ≤24.9)	81 (37%)
Overweight (≥25 to ≤29.9)	54 (24%)
Obese class 1 (≥30 to ≤34.9)	47 (21%)
Obese class 2 (≥35 to ≤39.9)	19 (9%)
Obese class 3 (≥40^2^)	17 (8%)
Smoker^*^	
Yes	24 (10%)
No	223 (90%)
Alcohol intake^*^	
Yes	145 (59%)
No	102 (41%)
Regular periods^*^	
Yes	184 (76%)
No	58 (24%)
Number of pregnancies^*^	1 ± 1
Number of children^*^	0 ± 1
Time trying to conceive^*^ (months)	32 ± 24
Less than 1 year	30 (12%)
1–2 years	83 (33%)
>2 years	77 (31%)
Not applicable^**^	30 (12%)
Unknown	30 (12%)
Previous fertility treatment	
None	180 (92%)
IVF	8 (3%)
Clomid	11 (4%)
IUI	1 (1%)

^*^Incomplete data set; ^**^Fertility preservation, Mayer Rokitansky Kuster Hauser (MRKH), on treatment for endometriosis, paralysis, presently single, same sex relationship, male partner known to be infertile.

IVF, *in vitro* fertilisation; IUI, intra uterine insemination.

### Habitual PA observations

Of the 229 women who fully and accurately completed the IPAQ-SF, approximately half of the participants achieved the aforementioned criteria for moderate levels of PA, while approximately a quarter of the participants achieved low or high levels(Table 2). Women who reported taking part in structured exercise (*n = *71, 28%) spent 172 ± 105 min/week doing this. On average, walking was performed on 5 ± 2 days/week for 61 ± 88 min/day. Among women who reported walking ≥10 min daily (46%, *n*  = 98), walking was performed 79±103 min/day. Only 7% (*n* = 15) of women reported walking for 0 min/week, while time spent sitting was 5 ± 4 h/day.

**Table 2:** Physical activity levels of the participants (*n*  = 229) determined using IPAQ-SF. Data are presented as *n* (%) or as mean ± s.d.

Physical activity outcomes	Levels
Physical activity level	
High	58 (25%)
Moderate	105 (46%)
Low	66 (29%)
Total physical activity (min/week)	87 ± 64
Total physical activity (MET-min/week)	1607 ± 1513
Vigorous activity (MET-min/week)	427 ± 669
Moderate activity (MET-min/week)	278 ± 535
Walking activity (MET-min/week)	902 ± 961

### Correlations

Analysis of all physical activity variables, measured using the IPAQ-SF, revealed no significant correlation with demographic variables including age, BMI, units of alcohol consumed, regular periods, or time trying to conceive (*P* > 0.05).

### Acceptability

Of the 234 women who responded to the question querying whether they would be interested in participating in future research investigating the effect of PA on fertility, 31.2% (*n = *73) answered yes. Among those who answered yes, women recorded the maximum amount of time they would be happy to delay fertility treatment if they took part in a future research trial (Table 3).

**Table 3:** Preferred maximum time of delay to fertility treatment among women interested in participating in future research (respondents, *n*  = 73).

Delay to fertility treatment	*n* (%)
1 month	28 (38)
3 months	8 (11)
6 months	6 (8)
12 months	12 (17)
Not sure	19 (26)

## Discussion

Habitual PA in women experiencing infertility is unknown. Furthermore, PA is neither currently considered during routine fertility care consultations nor are there any clinical guidelines available to advise women aiming to conceive regarding safe frequency, duration, intensity, and modes of PA. The aim of the current study was, therefore, to determine habitual PA in a sample of women attending the OSI clinic. Our study found a wide variation of PA levels and types, with some women reporting performing vigorous exercise on ≥5 days per week (8%, *n*  = 17), and a large proportion of women (29%, *n*  = 66) stating that they do no moderate or high-intensity activities across the whole week, while most women reporting no structured exercise (72%, *n*  = 179).

Evidence is available from large prospective observational studies informing that vigorous PA could be detrimental to fecundability in a population of women, with a normal BMI, not seeking fertility treatment (Wise *et al.* 2012, McKinnon *et al.* 2016), and change in menstrual cycle is also seen in women participating in vigorous exercise (Bullen *et al.* 1985, Ellison & Lager 1985, De Souza *et al.* 1998). Despite this, there is a lack of robust evidence and guidelines relating to women seeking fertility treatment to advise on how best to incorporate PA into their daily routine. Importantly, a considerable proportion of women in our cohort were outside the healthy BMI range (63%, *n*  = 138), which has consistently been demonstrated within the literature to decrease fecundability, as well as increase the risk of miscarriage and pregnancy complications (Satpathy *et al.* 2008, Silvestris *et al.* 2018, Cozzolino *et al.* 2021). Therefore, in the context of increasing rates of women seeking fertility support, it is important to understand whether habitual PA could act as a non-pharmacological aid, with or without weight loss, and ensure that we are providing our patients with the best evidence-based care.

A recent systematic review has shown that PA may improve pregnancy rates in women with reproductive health problems, but the mode, intensity, frequency, and duration of optimal PA intervention, and the role of PA independent of weight loss, remains unclear (Mena *et al.* 2019). In a cohort of obese women undergoing IVF, those who performed regular PA prior to assisted reproduction experienced higher success rates, (39% vs 16% in sedentary women, *P* = 0.002) and improved live birth rates (24.4% vs 7.4%, *P* = 0.004) compared with women who did not, irrespective of weight loss (Palomba *et al.* 2014). This evidence provides encouraging support for the use of PA recommendations/guidelines for obese women, who are aiming to conceive. Furthermore, a 2002 questionnaire-based prospective cohort study, originally designed to examine the association between lifestyle (smoking/exercise) and diet with breast cancer and other major diseases, found that each hour of vigorous activity undertaken per week was associated with a 7% lower relative risk of ovulatory infertility (5% on adjustment of BMI) (Rich-Edwards *et al.* 2002). This is in contrast to other studies examining ovulatory infertility and exercise (Bullen *et al.* 1985, Ellison & Lager 1985, De Souza *et al.* 1998) and should perhaps be interpreted with caution as the outcomes do not relate to the original question asked within the study.

PCOS is recognised as the leading cause of anovulatory infertility and affects 12–18% of women of reproductive age (March *et al.* 2010), with obesity prevalent in 40–88% of affected women (Balen *et al.* 1995, Azziz *et al.* 2004, Lim *et al.* 2012). Lifestyle modification, including PA, has been seen as a first-line approach in PCOS management (Teede *et al.* 2011). In a systematic review, women with PCOS were shown to have higher pregnancy rates (RR: 1.59, 95% CI: 1.06, 2.38; five studies) and live birth rates (RR: 2.45, 95% CI: 1.24, 4.83; two observations) in PA intervention groups, compared with non-intervention controls. Further analysis showed no significant differences in improving menstrual regularity, ovulation, and conception rates between the PA intervention and comparison groups (Mena *et al.* 2019). Despite the high prevalence of PCOS, and the indisputable link with fertility, no robust evidence is currently available to recommend the optimum mode and volume of PA (Harrison *et al.* 2011, Lim *et al.* 2019, Dos Santos *et al.* 2020).

Over a quarter of the women in our cohort did low levels of PA and a quarter did high levels of PA, both could interestingly be impacting their fertility. A study looking at sedentary behaviour and idiopathic infertility showed that sedentary behaviour (OR: 3.61; 95% CI, 1.58, 8.24) was shown to be associated with infertility, but PA was not associated with fertility (Foucaut *et al.* 2019). Conversely, evidence is also available demonstrating that high-intensity exercise is detrimental to fertility, causing a decreased length of the luteal phase, anovulatory cycles, and eventually, amenorrhoea (Bullen *et al.* 1985, Ellison & Lager 1985, Green *et al.* 1986, De Souza *et al.* 1998, Hakimi & Cameron 2017, Huhmann 2020, Sophie Gibson *et al.* 2020). A large population-based health survey reported that increased frequency, duration, and intensity of PA were associated with increased subfertility. Women who were active on most days of the week were 3.2 times more likely to have fertility problems than inactive women, with exercising to exhaustion associated with 2.3 times the odds of fertility problems, vs low-intensity exercise (Gudmundsdottir *et al.* 2009). High-intensity exercise has also been associated with a 5-fold increase in IVF cycle cancellation, a 2.5-fold increase in failed implantation, a 30% lower chance of successful pregnancy, and a 50% reduction in live births compared with women who reported no regular PA in a large prospective study (Morris *et al.* 2006). A recent systematic review investigating the effect of exercise on ovulation showed an increased risk of anovulation in extremely heavy exercisers (>60 min/day), but vigorous exercise (30–60 min/day) was associated with a reduced risk of anovulatory infertility (Hakimi & Cameron 2017).

Our study is novel, since we elicit the habitual PA of women who attend the OSI clinic and do not provide a prescribed intervention assuming that one intervention would suit all women. We included all women regardless of their BMI or any previously diagnosed pathology ensuring a representative sample of women who struggle with fertility and attend the OSI clinic. Unfortunately, questionnaires have been reported to overestimate PA in women and, as such, objective measurements have been recommended (Bell *et al.* 2013). However, they remain valuable to define the types and profiles of activity (Craig *et al.* 2003) and would provide preliminary information pertinent to this cohort of patients, thus were selected as the best methodological approach.

PA is an easily modifiable, affordable, and feasible lifestyle choice, which is currently ignored in the fertility setting and no guidance is available yet to inform either clinicians or their patients. This low-risk and low-cost lifestyle choice has the potential to improve fertility without compromising the general wellbeing of the patient. We would like to add a caveat that a ‘one size fits all’ level of PA cannot be advocated, and a personalised approach, with a holistic take on lifestyle, not just on smoking and alcohol but also optimising the appropriate level/types of PA should be recommended to each woman seeking fertility treatment. We already appreciate that PA reduces weight, but in those who do not need to reduce weight, the role of PA in improving their fecundity requires further study. The sole focus on PA for weight loss, rather than the effects of PA on reproductive function itself, has resulted in the current lack of studies comparing types of PA, intensity, and their impact on female fertility. This precludes the efficient and effective use of PA as a therapeutic modality in the management of infertility. An adequately powered, optimally designed, clinical trial assessing the role of PA in altering fecundability is urgently needed to evaluate the public health impact of that approach and to gain insight into the biological mechanisms of PA on reproductive function. This is especially pertinent in the era of unknown and unfair funding for fertility treatment across different regions and countries on a background of intense interest among young people in PA and exercise training.

## Declaration of interest

The authors declare that there is no conflict of interest that could be perceived as prejudicing the impartiality of the research reported.

## Funding

This work was supported by the Wellbeing of Women (grant numbers RTF510 (N T and D K H) and RG1073 (D K H) and RG2137 (D K H)). N T is supported by an Academic Clinical Lectureship from the National Institute of Health Research. H A L is supported by the Hewitt Fertility Centre/Liverpool Women’s Hospital NHS Trust.

## Author contribution statement

Conceptualisation and ethical approval N T and D K H; N T, H A L, E O and E S collected the data, N T, M F, V S, and D K H analysed and interpreted the data and wrote the first draft of the manuscript. All authors have read and agreed to the final version of the manuscript.
